# Whole-body transcriptome analysis provides insights into the cascade of sequential expression events involved in growth, immunity, and metabolism during the molting cycle in *Scylla paramamosain*

**DOI:** 10.1038/s41598-022-14783-w

**Published:** 2022-07-06

**Authors:** Lei Liu, Xiao Liu, Yuanyuan Fu, Wei Fang, Chunlin Wang

**Affiliations:** 1 School of Marine Sciences, Ningbo University, No.169, Qixing South Road, Meishan Port District, Beilun District, Ningbo, 315832 Zhejiang China; 2 Ningbo Institute of Oceanography, Ningbo, 315832 China

**Keywords:** Transcriptomics, High-throughput screening

## Abstract

The molecular mechanisms underlying the dynamic process of crab molting are still poorly understood at the individual level. We investigated global expression changes in the mud crab, *Scylla paramamosain*, at the transcriptome level and revealed a cascade of sequential expression events for genes involved in various aspects of the molting process using whole-body sequencing of juvenile crabs. RNA-sequencing (RNA-seq) produced 139.49 Gb of clean reads and 20,436 differentially expressed genes (DEGs) among different molting stages. The expression patterns for genes involved in several molecular events critical for molting, such as cuticle reconstruction, cytoskeletal structure remodeling, hormone regulation, immune responses, and metabolism, were characterized and considered as mechanisms underlying molting in *S. paramamosain*. Among these genes, we identified 10,695 DEGs in adjacent molting stages. Gene Ontology (GO) and Kyoto Encyclopedia of Genes and Genomes (KEGG) analyses showed that significantly enriched pathways included structural constituents of cuticle, binding and chitin metabolic processes, steroid hormone biosynthesis, insulin resistance, and amino sugar metabolic processes. The expression profiles of 12 functional genes detected via RNA-seq were corroborated via real-time RT-PCR assays. The results revealed gene expression profiles across the molting cycle and identified possible activation pathways for future investigation of the underlying molecular mechanisms.

## Introduction

Molting is an important and ongoing process of physiological change that occurs throughout the life history of all crustaceans, with periodic shedding of the old cuticle and the subsequent reconstruction of a new rigid exoskeleton^1,2^. Several factors are common to the molt cycle in all crustaceans, including the transcription of cuticular genes, chitin synthesis, exoskeleton reconstruction, and hormone regulation^3,4^. Previous studies have made significant advances in furthering the understanding of the molting cycle concerning growth characteristics, material changes, energy metabolism, and hormone regulation at the ecological and physiological levels^5–9^. Moreover, recent studies have explored molting-related genes and their effects on protein synthesis^10^, chitin metabolism^11^, and muscle hydration control^12^ during the molting cycle. In addition, great progress has been made in elucidating molting in the last decade by focusing on the effects of environmental factors, the immune system, and endocrine regulation molting, and by conducting gene expression analysis^13–28^. However, the molecular events and mechanisms associated with the dynamic process of molting remain poorly understood in crustaceans, especially in crabs. *Scylla paramamosain* (Crustacea:Decapoda:Portunidae), commonly known as the mud crab, is widely distributed throughout the temperate, subtropical, and tropical areas of the Pacific Ocean and Indian Ocean. This species is also cultivated in many countries. *S. paramamosain* undergoes molting up to 18 times during its life cycle, including during larval development, during growth-related molting, and in adult reproductive molting^29^. The economic significance of this species makes the mud crab a valuable basis for exploring the genes involved in the molting cycle. Several researchers have described the effects of environmental factors and hormone-related genes on the molting cycle of *S. paramamosain*. A previous study evaluated the effects of temperature, salinity, starvation, and autotomy on the molting of early juvenile mud crabs and found that changes in ecdysone receptor gene expression levels appeared to play an important role in regulating the molting process^30^. Molt-inhibiting hormone (MIH) genes and the crustacean hyperglycemic hormone (CHH) genes that serve as key regulators of hormones controlling the molting process in crustaceans have been cloned, and their distribution in different tissues during the molting cycle of the mud crab have been explored^31,32^. Although the expression profiles of the chitinase gene family and the Na^+^/K^+^/2Cl^−^ cotransporter in mud crabs have been explored during molting^29,33^, the knowledge of the molecular mechanisms underlying molting remains limited. Therefore, it is important to elucidate the mechanisms underlying crab molting and to characterize the cascade of sequential expression events in molt-related genes involved in various aspects of the molting process. This will facilitate further investigation of functional genomics of *S. paramamosain* and other closely related species.

The present study attempted to provide a comprehensive transcriptional landscape for further studies of the molecular mechanisms governing the molting cycle process in *S. paramamosain* using RNA-sequencing (RNA-seq). This resource and the associated findings will provide insights into the biological processes of the molting cycle in this species and will help to improve the performance of mud crab in aquaculture.

## Results

### Illumina sequencing and transcriptome assembly

**Figure 1:**
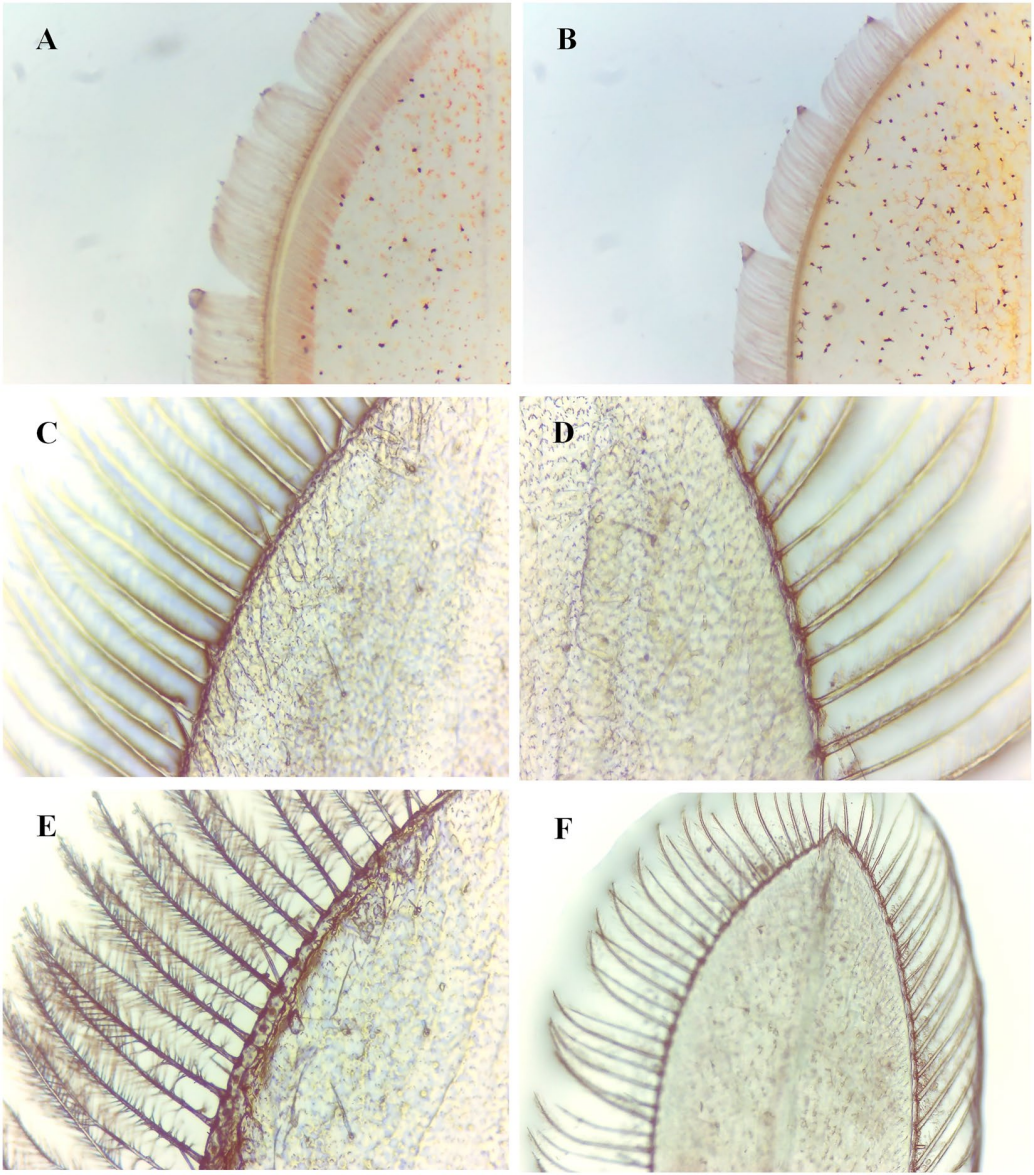
Morphological changes of crab uropods during molting stages under a light microscope (**A**,**B** are shown at ×50 magnification; **C**–**F** at ×400 magnification). The observed physical characteristics of each stage are as follows: Pre-molt stage (**A**): a noticeably wider clear zone between the setal cones and the epidermis, and new setae are fully generated; post-molt stage (**B**): soft and delicate setae and dense connective tissue; Intermolt stage (**C**): setae harden and the absence of setal cones; (**D**) fully-spread epidermis; (**E**) a clear margin of epidermal tissue at the base of the setal cones; (**F**) a narrow zone between the setal cones and the epidermis; setal cones arise.

**Figure 2:**
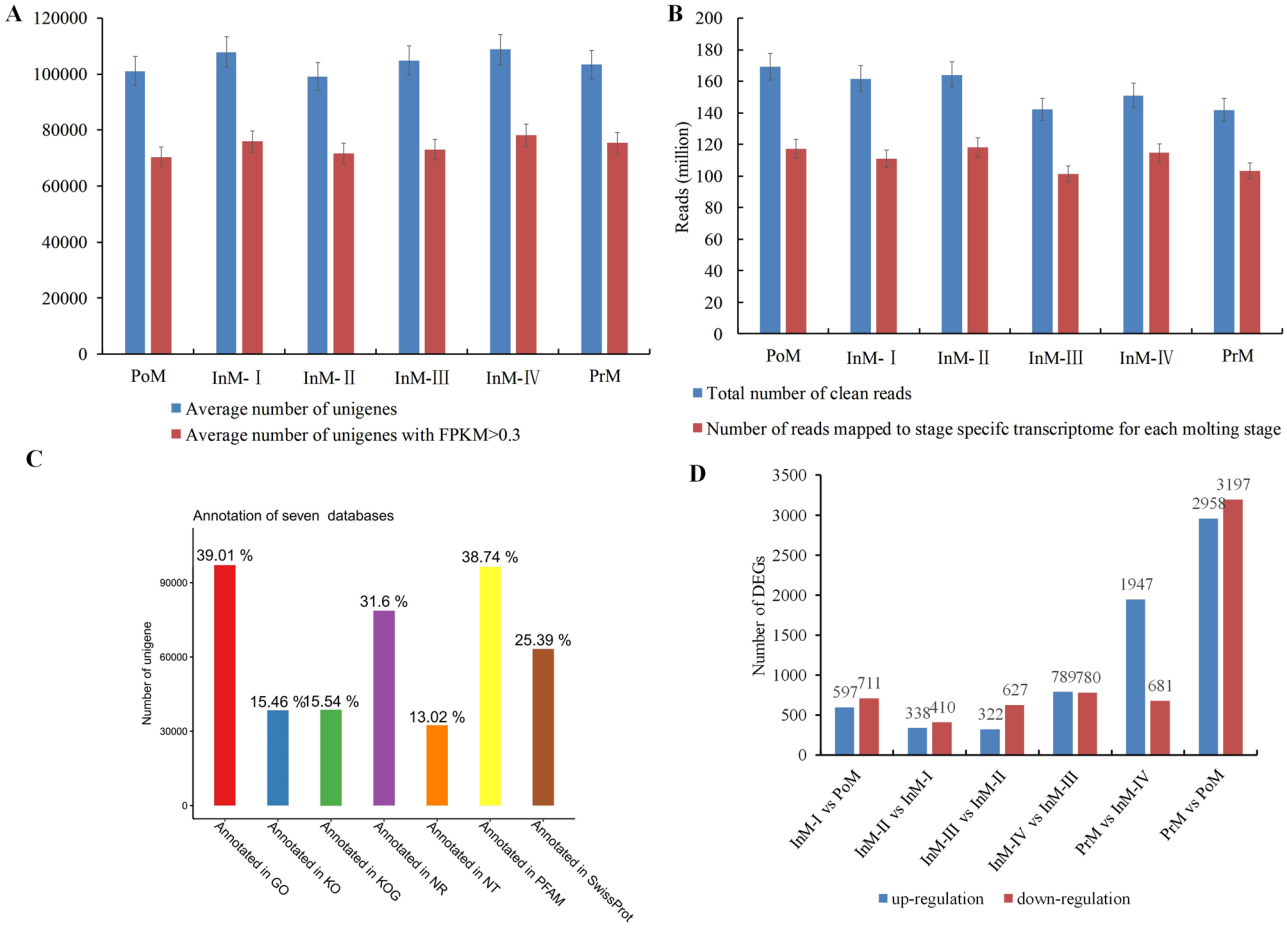
Assembly, mapping, and annotation of transcriptomes and differentially expressed genes (DEGs) between six comparisons of adjacent molting stages in *Scylla paramamosain*. (**A**) Average number of unigenes and number of genes with fragments per kilobase of exon per million mapped fragments (FPKM) ≥ 1; (**B**) total number of reads, number of reads mapped to the reference transcriptome, and number of reads mapped to the stage-specific transcriptome for each molting stage; (**C**) summary of unigenes annotated in different databases. *NR* NCBI non-redundant protein sequences, *NT* NCBI non-redundant nucleotide sequences, *Pfam* protein family, *Swiss-Prot* a manually annotated and reviewed protein sequence database, *KOG* Clusters of Orthologous Groups of proteins, *GO* Gene Ontology, *KEGG* Kyoto Encyclopedia of Genes and Genomes database, *All* total number of unigenes that were successfully annotated in at least one database; (**D**) DEGs between six comparisons of adjacent molting stages (InM-I vs PoM, InM-II vs InM-I, InM-III vs InM-II, InM-IV vs InM-III, PrM vs InM-IV, and PrM vs PoM). The number of up-regulated and down-regulated genes between comparisons is given. The x-axis indicates adjacent stages in comparisons. The y-axis indicates the number of DEGs.

The assembly generated 953,784,150 raw reads from 18 samples taken at the six molting stages (Fig. 1). A draft assembly of the *S. paramamosain* transcriptome was constructed with 139.49 Gb of high-quality filtered short-read Illumina sequence data (Supplementary Tab. S1). All transcriptome data are available in the National Center for Biotechnology Information (NCBI) Short Read Archive (SRA) database under accession number PRJNA687923. Finally, an average of 51,664,527 clean reads were obtained, with the Q20 of all samples being greater than 97% (Supplementary Tab. S1). The number of unigenes with fragments per kilobase of exon per million mapped fragments (FPKM) > 0.3 ranged from 70,319 to 78,247 among the eight molting stages (Fig. 2A). Over 100 million clean reads were mapped to the stage-specific transcriptome (Fig. 2B). More than 70% of the reads were mapped to the filtered reference transcriptome and the stage-specific corresponding transcriptome (Fig. 2B, Supplementary Tab. S1).

In the assembly, 350,531 transcripts (each with more than 200 bp) with a total length of 339,582,267 bp were obtained. A large portion of the transcripts were between 200 and 500 bp (Table 1). The transcripts had an N50 size of 2224 bp, a mean transcript length of 969 bp, a maximum transcript length of 33,949 bp, and a mean unigene length of 1257 bp (Table 1).

**Table 1:** *Scylla paramamosain* transcriptome statistics.

Length	Transcripts	Unigenes
200–500 bp	208,438	107,506
500–1 kbp	58,411	57,615
1 k–2 kbp	38,919	38,866
> 2 kbp	44,763	44,757
Total number	350,531	248,744
Mean length	969	1,257
Max length	33,949	33,949
N50	2224	320
N90	2505	455
Total nucleotides	339,582,267	312,762,368

#### Transcriptome annotation

The NCBI non-redundant proteins (NR), NCBI Nucleotide (Nt), Swiss-Prot, Pfam, Clusters of Orthologous Groups (KOG), Gene Ontology (GO), and Kyoto Encyclopedia of Genes and Genomes (KEGG) databases were used for unigene annotation. A total of 248,744 genes were successfully annotated through alignment to one or more of the databases (Supplementary Tab. S2). Of these 248,744 unigenes, 78,617 (31.6%) matched to known proteins in the NR database (Supplementary Tab. S3), and 32,401 (13.02%) matched to putative homologues in the Nt database. Among all annotated databases, the largest proportion of annotation hits (39.01%) was obtained with the GO database, followed by the Pfam database (38.74%, Supplementary Tab. S2, Fig. 2C). In addition, the KOG database provided annotation for 38,663 (15.54%) unigenes, the Swissprot database confirmed matches for 63,177 (25.39%) unigenes, and 38,478 (15.46%) unigenes found putative homologues in the KEGG database (Supplementary Tab. S2, Fig. 2C). Of the unigenes, 120,837 (48.57%) exhibited a positive match against at least one of the seven databases, whereas 13,062 (5.25%) had the best BLAST matches to proteins in all four databases.

The similarity analysis between *S. paramamosain* unigenes and NR protein databases was conducted using BLAST matches with a cut-off E-value of 1.0E−5 (Supplementary Fig. S1A). For the main species distribution matched against the NR database, only 10.2% of the matched unigenes showed similarities to *Zootermopsis nevadensis*, followed by *Daphnia pulex* (5.4%), *Tribolium castaneum* (3.2%), *Stegodyphus mimosarum* (2.5%), *Crassostrea gigas* (2.2%), and others (76.4%) (Supplementary Fig. S1B). There were 1.5%, 34.3%, 44.2%, 16.3%, and 3.6% putative proteins showing 18–40%, 40–60%, 60–68%, 80–95%, and 95–100% similarity, respectively, to the known proteins in the NR protein database (Supplementary Fig. S1C).

In the present study, a total of 97,051 unigenes were assigned to 56 sub-categories of GO terms belonging to the following three main categories: cellular component, molecular function, and biological process, which included 20, 10, and 26 subcategories, respectively (Supplementary Fig. S2A). For cellular components, cell (38,969 genes), cell part (38,968 genes), organelle (28,306 genes), membrane (25,539 genes), and macromolecular complex (24,009 genes) represented most of the categories. Binding (48,876 genes), catalytic activity (35,003 genes), transporter activity (11,160 genes), structural molecule activity (5699 genes), and molecular transducer activity (4038 genes) represented the activity categories of molecular function. Cellular process (59,345 genes), metabolic process (48,887 genes), single-organism process (43,713 genes), biological regulation (25,401 genes), and localization (23,965 genes) represented a high percentage of the biological process category. The results of KEGG pathway analysis showed that 38,478 unigenes were annotated into five main categories and 32 sub-categories (Supplementary Fig. S2B). Among the main categories, organismal systems had the largest number of unigenes (13,843 unigenes), followed by metabolism (7698 unigenes), genetic information processing (8824 unigenes), cellular processes (7956 unigenes), and environmental information processing (6318 unigenes). Among the 32 sub-categories, translation was the largest group with 5668 unigenes, followed by signal transduction (5365 unigenes). The smallest group, containing only 53 unigenes, was biosynthesis of other secondary metabolites. As a consequence, 38,663 annotated genes in KOG were grouped into 26 KOG categories, among which the cluster for “translation, ribosomal structure and biogenesis” (5704 unigenes) represented the largest group, followed by “signal transduction mechanisms” (5570 unigenes), “general function prediction only” (5297 unigenes), “post translational modification, protein turnover, chaperones” (4161 unigenes), and “cytoskeleton” (2513 unigenes); “nuclear structure” (144 unigenes) and “cell motility” (50 unigenes) represented the smallest groups (Supplementary Fig. S2C).

#### Expression profile of differentially expressed genes (DEGs) across the molting cycle of *S. paramamosain*

A total of 20,436 DEGs were identified across the six molting stages (Tab. S4). The expression levels of DEGs during the molting cycle were divided into 22 categories based on K-means clustering, which revealed the different expression patterns of DEGs among the six molting stages (Supplementary Fig. S3). The largest group, cluster 3, contained 3028 DEGs with gene expression levels decreasing during the InM-I vs PoM and InM-III vs InM-II transitions. Other clusters containing over 1000 members were cluster 10 (1458 DEGs), cluster 11 (1474 DEGs), cluster 12 (1038 DEGs), cluster 16 (1091 DEGs), cluster 19 (2739 DEGs), and cluster 20 (1528 DEGs) (Supplementary Fig. S3).

**Figure 3:**
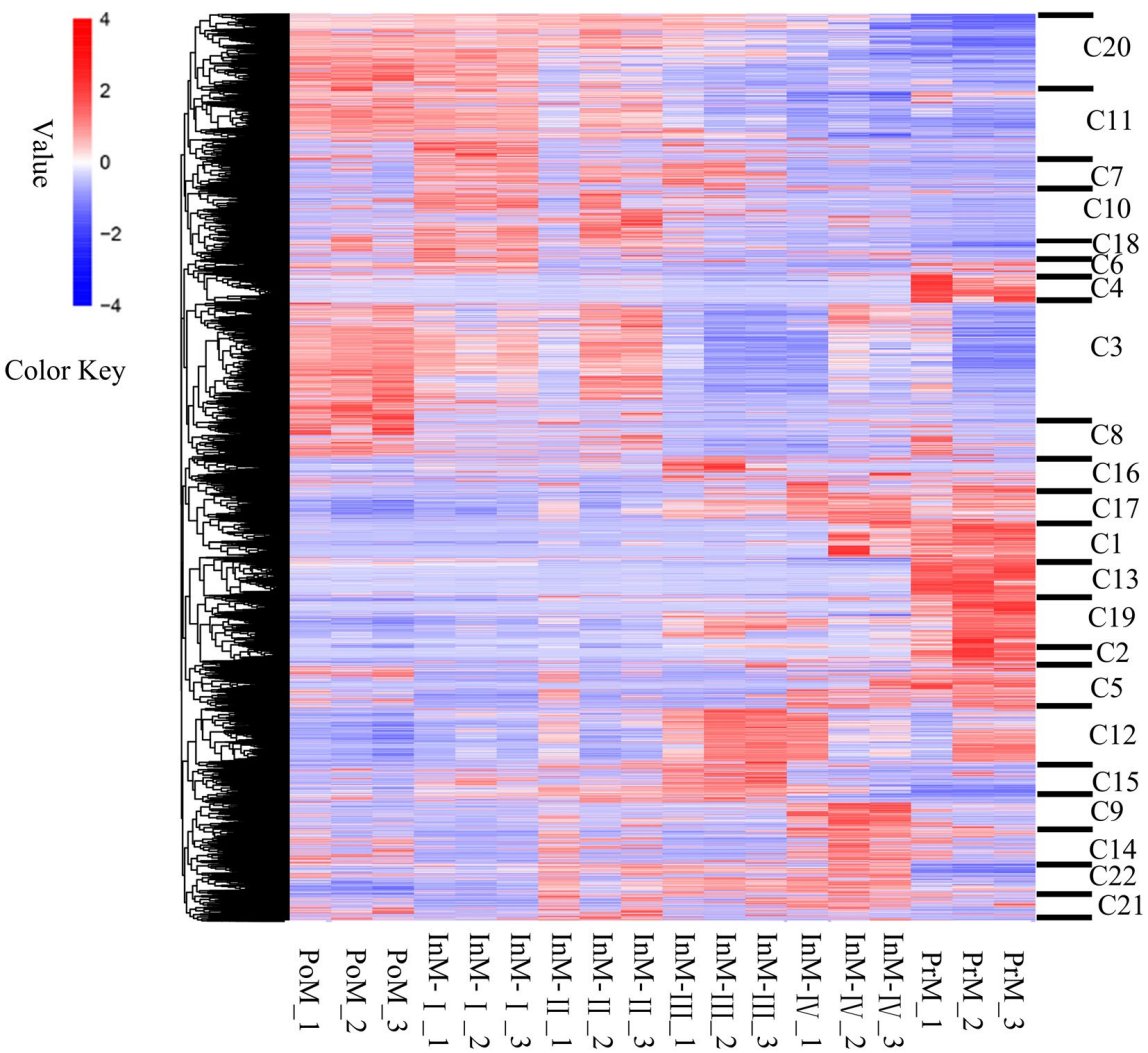
Heat map of DEGs clustered in two ways: molting stages and GO categories. Each stage has three replicate samples. C1–C22 functional clusters of DEGs. Color key value indicates the fragments per kilobase of exon per million mapped fragments (FPKM) fold change.

GO enrichment analysis (FDR < 0.005) of these DEGs revealed the enrichment of multiple terms of biological processes, cellular components, and molecular functions associated with the molting cycle (e.g., structural constituents of cuticle, amino sugar metabolic processes, chitin metabolic processes, and hormone receptor binding) (Fig. 3). Profiling gene expression and GO enrichment analysis of DEGs showed that genes in clusters 3, 7, 8, 10, 11, 18, and 20 were predominantly up-regulated in the molting stages InM-I, InM-II, and PoM, and were down-regulated in the InM-III, InM-IV, and PrM stages, which were mainly associated with “myosin complex”, “motor activity”, and “actin cytoskeleton”; and “structural molecule activity”, “structural constituent of cuticle”, and “chitin binding”; and “amino sugar metabolic process”. Conversely, the DEGs in clusters 12, 16, 17, and 19 were predominantly down-regulated in the molting stages InM-I, InM-II, and PoM, and were up-regulated in InM-III, InM-IV, and PrM, which were mainly associated with “glucosamine-containing compound metabolic process”, “amino sugar metabolic process”, “GTP binding”, “oxidoreductase activity”, “guanyl ribonucleotide binding”, “3-beta-hydroxy-delta5-steroid dehydrogenase activity”, “C21-steroid hormone metabolic process”, and “regulation of hormone levels”. Interestingly, DEGs in clusters 2, 4, and 13 were only up-regulated in stage PrM, and these DEGs were strongly enriched in “structural constituent of cuticle”, “structural molecule activity”, “chitin metabolic process”, “glucosamine-containing compound metabolic process”, and “amino sugar metabolic process”. Moreover, DEGs in clusters 1 and 16 showed that the expression of genes increased with the number of days following molting. The details of the top 30 GO categories of the 22 clusters are shown in Supplementary Tab. S5.

#### Expression profiles of DEGs associated with growth, immunity, and metabolism among molting stages

The molting of crustaceans is accompanied by structural changes and material metabolism in various tissues of the body, among which muscles and the carapace undergo the most dramatic changes during the molting cycle. In this study, dozens of DEGs associated with carapace, muscle, and hemolymph were identified (Supplementary Tab. S6). Of these DEGs, many transcripts were found to be related to the composition and organization of the shell in crustaceans, including arthrodial cuticle protein, BCS, calcified cuticle protein, chitin-binding protein, chitin deacetylase, chitinase, cuticle protein, cuticular protein, early cuticle protein, and gastrolith protein. Members of different gene families exhibited various characteristics during the molting cycle. For example, chitinase 1 and 3 showed up-regulated expression trends from PoM to InM-III, but both decreased at stage InM-IV. Moreover, all four early cuticle protein family members had down-regulated expression patterns from PoM to InM-IV and then increased suddenly at stage PrM. The muscle- and skeleton-related genes included many aspects of muscle formation and function from structural protein to muscle contraction such as actin, muscle LIM protein, muscle and skeletal receptor tyrosine protein kinase, cardiac muscle actin, and myosin. In addition, six genes were found to be associated with hemocyte homeostasis and composition, including cryptocyanin, hemocyte kazal-type proteinase inhibitor, hemocyte homeostasis associated protein, hemocyanin, and spz3 (Supplementary Tab. S6). Interestingly, some DEGs showed great changes in expression. For example, the FPKM values of cuticle protein CUT2 increased more than 10,000-fold from InM-I to InM-II, but decreased from InM-III onwards, reaching zero at stage PrM. Additionally, the FPKM values of cuticle proprotein proCP and cuticular protein 15 decreased more than 3000-fold from InM-III to InM-IV, and the same pattern was found for gastrolith protein 30, with a more than 3000-fold decrease from InM-II to InM-III.

This study identified 21 important hormone-related genes that may be directly or indirectly involved in controlling the crustacean molting cycle (Supplementary Tab. S6). Of these genes, the expression levels of ecdysis-triggering hormone receptor subtype-B and ecdysone-induced protein showed characteristic patterns of down-regulation during the entire molting cycle from PoM to PrM. The expression of ecdysteroid-regulated protein was up-regulated from PoM to InM-II, and then declined from InM-III to PrM. Estrogen sulfotransferase was up-regulated from PoM to InM-IV. but increased suddenly at stage PrM, and the same pattern was found for estradiol 17-beta-dehydrogenase 8 and juvenile hormone epoxide hydrolase (Table 2).

**Table 2:** Expression levels of molting regulation-related hormone genes.

Factor name	Gene number	Average FPKM value					
		PoM	InM-I	InM-II	InM-III	InM-IV	PrM
Androgen-induced protein 1	1	2.41	1.57	3.21	7.69	8.67	4.9
Crustacean hyperglycemic hormone	1	0.94	0.19	0.55	0.28	4.22	5.18
Ecdysis triggering hormone receptor subtype-B	2	13.42	8.05	7.63	4.43	4.14	3.14
Ecdysone receptor	4	1.91	2.37	1.75	2.22	1.38	1.34
Ecdysone induced protein	4	4.01	0.67	0.56	0.14	0.1	0.09
Ecdysteroid regulated protein	4	208.13	368.08	471.96	448.12	56.74	28.86
Estradiol 17-beta-dehydrogenase 8	4	1.75	4.43	14.39	18.52	109.8	14.37
Estradiol receptor-like protein 1	1	1.01	0.04	0.13	0	0	0.04
Estrogen sulfotransferase	7	4.14	5.74	13.03	20.13	35.54	6.71
Farnesoic acid *O*-methyltransferase long isoform	1	9.29	12.56	12.53	75.18	51.38	62.54
Female sterile homeotic protein	2	8.17	4.4	7.6	2.91	5.51	2.41
Hormone receptor 4	1	0.42	0.49	0.34	0.06	0.03	0.21
Hormone receptor 4 isoform X	4	14.79	9.85	7.6	2.2	0.65	9.28
Hormone receptor 78	2	6.87	6.52	7.5	3.53	1.24	0.63
Hormone receptor hr3	3	0.27	0.12	0.17	0.18	0.21	21.81
Juvenile hormone acid methyltransferase	2	2.88	0.63	2	0.14	1.13	0.81
Juvenile hormone epoxide hydrolase	1	13.26	25.85	36.46	52.27	153.99	33.96
Lutropin-choriogonadotropic hormone receptor	2	0.77	0.12	0.66	0	0.54	0.08
Molting fluid carboxypeptidase A precursor	2	0.24	0.87	0.06	0.05	1.05	8.1
Vitelline membrane outer layer 1-like protein	2	80.75	99.5	93.34	153.24	139.87	156.19
Vitellogenin	1	0.15	0.08	3.38	0.03	0.02	7.11

To identify the possible factors involved in the molting process, this study also investigated the expression profiles of members of the immune system and metabolism during molting. There were 21 and 28 genes found to be related to the immune system and metabolism, respectively. Of the immune-related genes, the C-type lectin receptor was down-regulated in PoM but up-regulated in PrM, and the same pattern was found for crustin 2, crustin 3, beta-thymosin 4, and beta-thymosin 5. Interestingly, most immune system genes reached their maximum expression levels in InM-II; these included C-type lectin domain family 4-member, C-type lectin-2, C-type lectin receptor, C-type lectin, crustin-2, crustin 4, and ALF1. Twenty-eight genes related to substance metabolism, storage, and transportation were detected, including four sugar transporters, one sterol regulatory element-binding protein, six serine proteinases, three serine proteinase inhibitor accases, six cytochrome P450s, three lipoprotein receptors, one lipid storage droplet protein, and one lipase (Supplementary Tab. S7).

#### GO and KEGG enrichment analysis of DEGs in adjacent molting stages

**Figure 4:**
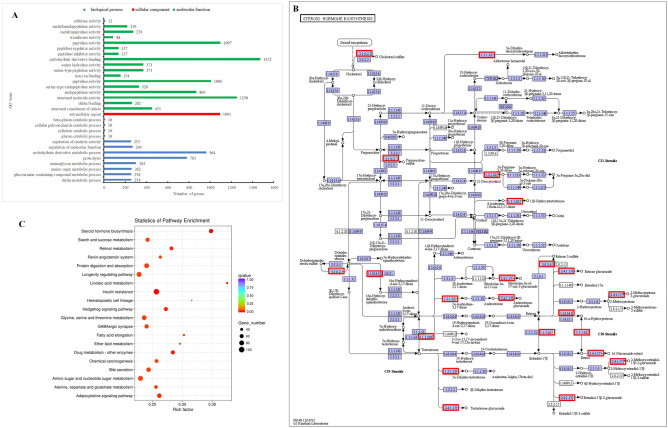
Gene ontology (GO) and Kyoto Encyclopedia of Genes and Genomes (KEGG) enrichment analysis of 10,695 DEGs among six comparisons of adjacent molting stages in the *Scylla paramamosain* molting cycle. (**A**) GO enrichment results; (**B**) statistics of pathway enrichment; (**C**) the networks of most significant enriched pathways of 10,695 DEGs (steroid hormone biosynthesis)^75^.

This analysis specifically investigated the interactions among DEGs between adjacent molting stages and their functioning in biological processes through GO and KEGG enrichment. A total of 10,695 DEGs between any two adjacent molting stages (i.e., InM-I vs PoM, InM-II vs InM-I, InM-III vs InM-II, InM-IV vs InM-III, PrM vs InM-IV, and PoM vs PrM) were identified (Supplementary Tab. S8, Fig. 2D). The 10,695 DEGs were further subjected to GO enrichment to predict their potential functions and to KEGG enrichment to obtain relevant metabolic pathways. To associate the different expression patterns of these DEGs with morphological and physiological changes during the molting cycle, GO enrichment analysis was performed and DEGs were sorted into different sub-categories that belonged to three main GO categories: biological processes, cellular components, and molecular functions. GO enrichment analysis revealed that structural constituents of the cuticle were the most significantly enriched pathway for 10,695 DEGs, accounting for 451 unigenes, followed by chitin-binding and chitin metabolic process (Supplementary Tab. S9, Fig. 4A). Notably, these top three enrichment pathways were all related to the molecular function and biological process of shell formation regulation during crab molting.

The 20 most enriched KEGG pathways are listed in Supplementary Tab. S10 and Fig. 4C. Among these, the 10 most important and representative pathways that were potentially relevant to gene interactions underlying the molting cycle included 616 unigenes that were matched to “steroid hormone biosynthesis” (63 unigenes), “insulin resistance” (102 unigenes), “linoleic acid metabolism” (33 unigenes), “drug metabolism-other enzymes” (60 unigenes), “retinol metabolism” (55 unigenes), “hedgehog signaling pathway” (60 unigenes), “adipocytokine signaling pathway” (67 unigenes), “protein digestion and absorption” (77 unigenes), “bile secretion” (69 unigenes), and “hematopoietic cell lineage” (30 unigenes) (Supplementary Tab. S10). In addition, the pathway of “steroid hormone biosynthesis” was the most representative pathway, with high hormone-related gene numbers in accordance with the characteristics of molting control; this is likely to be useful in future investigations focusing on their functions in the molting cycle of *S. paramamosain* (Fig. 4B).

In the comparison of InM-I vs PoM, 597 DEGs were up-regulated and 711 DEGs were down-regulated. These were sorted into 2585 GO sub-categories with six significantly enriched sub-categories (corrected P value < 0.05) of amino sugar metabolic processes, structural constituents of cuticle, chitin metabolic processes, glucosamine-containing compound metabolic processes, chitin binding, and viral release from host cell (Fig. 2D, Supplementary Fig. S4). In the comparison of InM-II vs InM-I, 338 DEGs were up-regulated and 410 DEGs were down-regulated; these were sorted into 2152 GO sub-categories with four significantly enriched sub-categories (corrected P value < 0.05) of structural constituents of cuticle, histidine biosynthetic processes, histidine metabolic processes, and imidazole-containing compound metabolic processes (Fig. 2D, Supplementary Fig. S4). A total of 322 DEGs were up-regulated and 627 DEGs were down-regulated in the comparison of InM-III vs InM-II, and no significantly enriched sub-categories (corrected P value < 0.05) were observed. In the comparison of InM-IV vs InM-III, 789 DEGs were up-regulated and 780 DEGs were down-regulated; these were sorted into 2506 GO sub-categories with 13 significantly enriched sub-categories (corrected P value < 0.05) (Fig. 2D, Supplementary Fig. S4). There were 1947 and 2958 up-regulated DEGs and 681 and 3197 down-regulated DEGs for the comparisons of PrM vs InM-IV and PoM vs PrM, respectively. The major significantly enriched sub-categories (corrected P value < 0.05) for the comparisons of PrM vs InM-IV and PoM vs PrM are shown in Supplementary Fig. S4.

**Figure 5:**
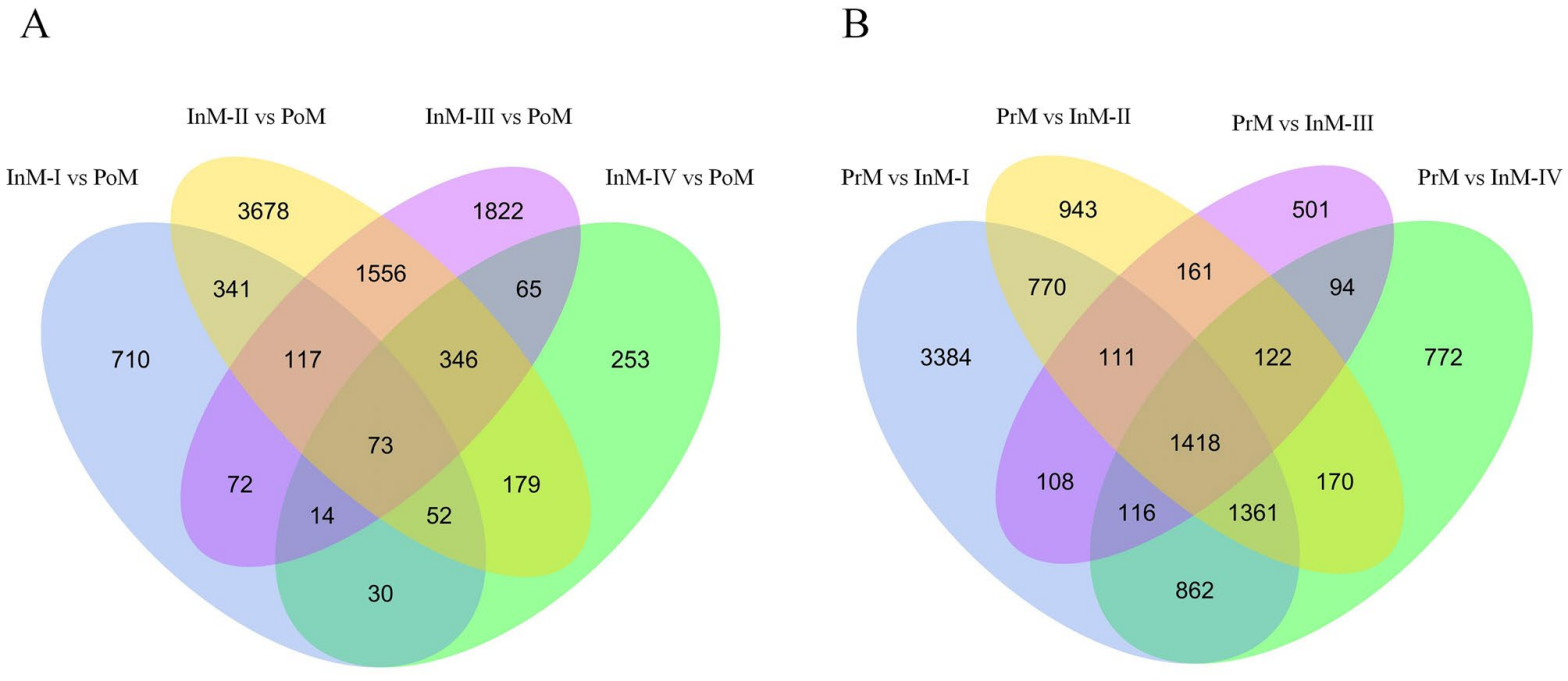
Venn diagram for the number of DEGs between comparisons of InM vs PoM and PrM vs InM. (**A**) InM-I vs PoM, InM-II vs PoM, InM-III vs PoM, and InM-IV vs PoM; (**B**) PrM vs InM-I, PrM vs InM-II, PrM vs InM-III, and PrM vs InM-IV.

To more precisely analyze the expression patterns of DEGs in the molting cycle, this study examined the expression profiles (Supplementary Tab. S11) and listed the top 10 GO enrichment categories of DEGs in pairwise comparisons among molting stages of PrM, PoM, and InM (Supplementary Tab. S12). Interestingly, Venn diagram analysis revealed a total of 73 and 1418 DEGs for pairwise comparisons between the four intermolt stages (InM-I, InM-II, InM-III, InM-IV) and PrM and PoM, respectively (Fig. 5). In InM vs PoM, 73 DEGs were sorted into the most enriched GO sub-categories associated with energy metabolism (e.g., adenosine kinase activity, AMP biosynthetic process, AMP metabolic process, and riboflavin synthase complex). In InM vs PrM, 1418 DEGs were sorted into the most enriched GO sub-categories associated with shell formation in the molting cycle (e.g., structural constituent of cuticle, structural molecule activity, chitin binding, and chitin metabolic process).

#### Quantitative reverse-transcription PCR (qRT-PCR) validation

**Figure 6:**
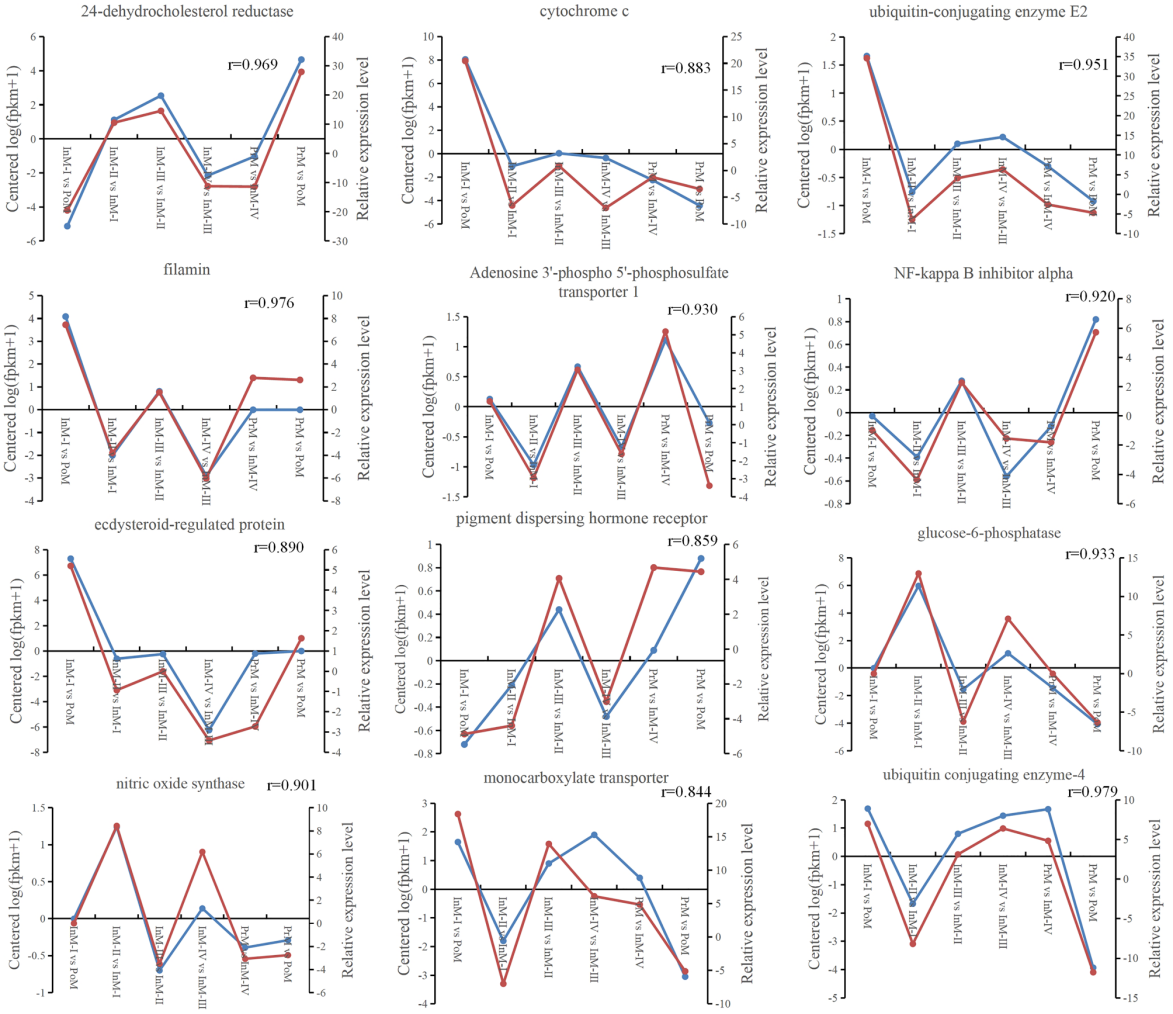
Expression profiles of 12 DEGs from RNA-sequencing (RNA-seq) (blue) and qRT-PCR (red) with alpha-tubulin as a reference gene in different molting stages.

For validation purposes, 12 representative genes were selected from the 22 clusters identified above and their expression levels were examined during the molting cycle using qRT-PCR. The qRT-PCR results confirmed the findings of RNA-seq, with correlation coefficient (r) values from 0.844 to 0.979 depending on the specific genes and reference genes (Fig. 6).

## Discussion

An *S. paramamosain* molt cycle-specific transcriptome derived from whole juvenile crabs, including individual organs such as the brain, gills, nerves, eyestalks, hepatopancreas, muscle, MO, and Y organs from all molt cycle stages, was developed in this study. The results showed some similarities to previous studies^3,4^ involving molting cycle transcriptome analyses in crustaceans; for example, some common factors related to the molting cycle were identified, such as the cuticular genes, chitin synthase, exoskeleton reconstruction, and hormone regulation. However, several differences exist between the results of the preset study and those of previous studies. These differences may be due to the materials and techniques used. The transcriptome database obtained using Illumina RNA-seq in this study was larger than those obtained by microarray-based analysis in other studies, and more genes were detected at each stage of the molting process in the present study. Second, previous studies mainly focused on the functional analysis of single organs during the molting cycle or on a specific stage, for example, focusing on muscle^34^, the hepatopancreas^4,33^, the eyestalks and heart^35^, or the mandibular organ and Y-organ^36^. Moreover, the present analysis revealed a cascade of sequential expression events of genes involved in various aspects of the molting process, including chitin degradation and synthesis, tissue development, hormone regulation, immune response, and energy metabolism. The present transcriptomic data are of high quality compared with previous studies and provide a comprehensive survey of each molting cycle stage of *S. paramamosain*.

Gene Ontology functional annotation analysis classified the predicted functions of the assembled unigenes. Cell binding and cellular processes were the most highly represented categories, consistent with transcriptome results of the whole claw muscles in *Eriocheir sinensis*^34^ and *Penaeus monodon*^35^. The results further demonstrated that the proliferation of epithelial tissues^37,38^, muscle^39^, and other development-related processes accompanying muscle and exoskeleton reconstruction were prominent during molting. Moreover, KEGG analysis demonstrated that the top enriched pathways were organismal systems instead of systems related to metabolism, as in previous studies^34,35^. These pathways included the circulatory system, environmental adaptation, the digestive system, the endocrine system, the immune system, and the nervous system. In addition, translation, ribosomal structure, and biogenesis were identified as the most enriched categories. Therefore, the present results suggest the biological functions and interactions of annotated unigenes involved in organ development, tissue reconstruction, and adjustment to environmental pressure during the molting cycle. These annotations provide a starting point for investigating the molecular mechanisms of molting-related genes in the molting cycle of *S. paramamosain*, as well as a valuable resource for further research into the specific functions and pathways of crustacean molting.

The molting cycle is a complex process that is normally divided into four stages: intermolt, premolt, ecdysis, and postmolt^40,41^. Overall, the clustering analysis and GO enrichment of DEGs across the molting cycle of *S. paramamosain* revealed the variational characteristics of molecular changes responding to morphological and physiological changes. For example, previous studies have indicated that postmolt (PoM) is a critical phase in the molting cycle of crustaceans, a period in which the animal is recovering from molting and during which its exoskeleton hardens quickly to avoid predation^4,34^. The most enriched GO terms of DEGs in the PoM, InM-I, and InM-II stages of *S. paramamosain* included “myosin complex”, “motor activity”, “actin cytoskeleton”, and “structural molecule activity”, revealing the gradual up-regulation of muscle-related genes and further indicating the important functions of these genes in muscle regeneration during the postmolt and intermolt periods^6,36,42^. Moreover, the up-regulation of genes in the premolt stage being enriched in the GO terms “3-beta-hydroxy-delta5-steroid dehydrogenase activity”, “C21-steroid hormone metabolic process”, and “regulation of hormone levels” demonstrated that the premolt stage, a critical phase in preparation for ecdysis, is controlled by hormonal regulation in the molting cycle of *S. paramamosain*.

Similar to other crustaceans, the growth of *S. paramamosain* is a stepwise process comprising periodic shedding, subsequent reconstruction of a rigid external exoskeleton and cuticle, and muscle growth to fill the new body during a molting cycle^3,43^. Some functional genes involved in the molting cycle of crabs were identified as associated with a series of biological processes in previous studies, such as cuticle reconstruction, cytoskeletal structure remodeling, protein synthesis, hormone regulation, immune response, and metabolism^4,29,31,33^. Among these processes, cuticle reconstruction and cytoskeletal structure remodeling have been considered to be essential for all four phases of the molting cycle^38^. More than 50 different cuticle-and cytoskeletal-related genes were identified as being differentially expressed across the molting cycle in this study. For example, chitinase digests the old exoskeleton to regenerate a new shell via chitin metabolism, which involves chitin synthase genes, chitinase genes, chitin deacetylase genes, and a number of genes whose gene products contain chitin-binding or other chitin metabolism-related domains^11,44,45^. Research on *Penaeus aztecus*^46^ and *P. monodon*^47^ has identified chitinase 2 as a direct factor in the molting process that is up-regulated in stage PrM and down-regulated in PoM. Moreover, a previous study in juvenile *Penaeus chinensis*^48^ has shown that the expression changes of chitinase 1 and 3 can be observed at all stages of the molting cycle. Finally, the present study identified six chitinases and found that they showed differential expression patterns in stages PoM, InM, and PrM, suggesting different physiological roles and modes of action of during the molting cycle. The chitin deacetylase gene is another chitin metabolism-related gene involved in the catalysis of the acetamido group in the N-acetyl-d-glucosamine units of chitin^49,50^^.^ To date, five classes of chitin deacetylase have been characterized, three of which were identified in this study (chitin deacetylase 1, chitin deacetylase 4, and chitin deacetylase 9). In insects, it has been demonstrated that the chitin deacetylase gene is involved not only in growth but also in the immune system^50,51^. However, few studies have characterized the chitin deacetylase genes or studied their function in the molting cycle of crustaceans. Most recently, the first study concerning the cDNA cloning of chitin deacetylase in a crab species was carried out in *Chionoecetes japonicus*^52^. The authors of the latter study hypothesized that chitin deacetylase was involved in ecdysis, as it was expressed only in the epidermis. Transcriptome analysis of molting-related tissues in *Cherax quadricarinatus*^11^ and *E. sinensis*^45^ revealed the up-regulated expression of chitin deacetylase during PoM compared to its levels during InM. In the present study, chitin deacetylase 1 and chitin deacetylase 4 had similar expression patterns to those found in previous studies, reaching the highest expression peaks at stage PoM, decreasing in the four periods of InM, and increasing again in PrM. In addition, the present study also observed similar transcriptional up-regulation of chitin synthase and chitin-binding protein genes, i.e., reaching the highest expression peaks at stage PoM, decreasing in the four periods of InM, and increasing again in PrM. The functions of these chitin metabolism-related genes in the molting cycle of *S. paramamosain* need further exploration in the future.

Dozens of cuticle protein-related genes have also been isolated, including calcified cuticle protein, cuticle protein AMP, cuticle protein CUT, and cuticular protein, some of which have extremely high expression levels and similar expression patterns as in chitin genes during the molting cycle. These include cuticle protein CUT2, cuticle protein BD1, cuticle proprotein proCP, and cuticular protein 15. The expression levels of these genes have been shown to be associated with cuticle calcification, by playing the role of inhibition or promotion in the molting process^53–55^. Gastrolith protein and gastrolith protein 30 showed up-regulation in the PoM stage and then decline during the four InM stages, followed by a sharp increase in the PrM stage. Gastrolith protein 10 clearly declined from PoM to PrM. The results suggest that the molting cycle process is complex, involving up- or down-regulation of these cuticle transcripts, indicating that the formation and/or repair of the exoskeleton may need them to operate separately. The new description of DEGs and the determination of their timing in different molting stages provide temporal markers for future studies of molting progress and regulation.

Cytoskeletal reconstruction is an important process for body recovery after molting^38^. However, factors participating in the enlargement of the cytoskeleton during the molting process are not yet completely clear. At the level of gene expression, this study identified a number of genes involved in osmotic regulation, muscle growth, and cytoskeletal structure remodeling that were up- or down-regulated at different molting stages. For example, three aquaporin genes were identified with lower expression levels during the PrM stage but higher levels during PoM and early InM, possibly due to the rapid water uptake that occurs after ecdysis. Previous gene expression studies in *Palaemon argentinus*^12^ and *S. paramamosain*^2^ have explored the role of aquaporin with Na^+^/K^+^-ATPase and Na^+^/K^+^/2Cl^−^ cotransporter in the uptake of water during the molting processes; these are osmoregulation-related genes with the same expression level trends in each of the molting stages in the present study. It can be inferred that aquaporin and other osmoregulation-related genes act synergistically to regulate osmotic pressure and water absorption after molting. Cytoskeletal reconstruction involves not only rapid water absorption but also expansion of the cytoskeleton to provide a scaffold and muscle to fill the new body^43^. The up-regulation of many cytoskeletal genes during early PrM and PoM was observed during the present study; these genes have been shown to be related to constituents of the microtubules, growth, and movement of muscle, including titin, tubulin, tropomyosin, and myosin. Among these cytoskeletal-related genes, titin binds to filamentous actin and provides elasticity to muscles in insects^56^, and tubulin is another important cytoskeletal group of proteins, making up the major constituents of microtubules^57^. Actin is a globular multi-functional protein that forms microfilaments and affects muscle plasticity in crustaceans^58,59^. As the major contractile protein in vertebrates and invertebrates, myosin has been identified to be associated with muscle atrophy during molting and is characterized by decreases in fiber width and myofibril cross-sectional areas, an increase in interfibrillar spaces, and degradation^39^. Hemocyanin is thought to play a role in cuticle formation and ecdysone transport during molting regulation and antigen non-specific immune defense by reversibly binding oxygen^60^ and displaying PO, ecdysone binding, and transportation activity^55,61^. Hemocyanin was significantly increased together with other hemolymph-associated genes during the late InM and PrM stages. Additionally, energy reserves, including glycogen and lipids, were also accumulated in the hemolymph for the next molt^34^.

The regulatory mechanism of molting is a complex process that includes a network of signals involving many hormone genes, such as the molt-inhibiting hormone (MIH) and crustacean hyperglycemic hormone (CHH) peptide families that control the molting process by inhibiting YO ecdysteroidogenesis and secretion^62,63^. Although it has been demonstrated that CHH is released from gut endocrine cells immediately before ecdysis in crustaceans, little is known regarding the stage-to-stage expression variation in molting, although this knowledge is clearly necessary for elucidating the significance of these hormones. In this study, CHH was up-regulated more than 15-fold during the InM-IV and PrM stages. During the same period, the expression levels of ecdysteroid-regulated proteins were sharply decreased by more than tenfold, which may have been caused by the suppression effect of CHH. The expression levels of other ecdysteroid-regulated genes also obviously decreased during the InM-IV and PrM stages, including ecdysis-triggering hormone receptor subtype-B, ecdysone receptors, and ecdysone-induced proteins. Similar expression results for CHH and ecdysteroid-related genes have been observed in other crustaceans just prior to ecdysis^38,64^. The same trend of up-regulated transcript levels was found in the vitellogenin gene (VG), which has been suggested to be an ecdysteroid-responsive gene in the molting process^65^. Farnesoic acid *O*-methyltransferase is another crustacean molting hormone-related category that may play a key role in growth regulation of crustaceans^3,66^. These enzymes were up-regulated from InM-III to PrM in the present study. In *P. chinensis*, a stage-specific expression profile revealed that the highest expression level of farnesoic acid o-methyltransferase occurred at the intermolt stage, implying that the conversion of farnesoic acid to methyl farnesoate may be involved in the onset of molting processes^66^. Moreover, many other growth and development-related hormone genes were also identified as differentially expressed during the molting cycle, including estradiol 17-beta-dehydrogenase 8, estrogen sulfotransferase, hormone receptor, juvenile hormone epoxide hydrolase, and lutropin-choriogonadotropic hormone receptor. Although the functions of these genes, which are potentially involved in hormone regulation, are still not well characterized, it appears that *S. paramamosain* produces a rather complex regulation profile of hormone genes as in other crustacean species. In summary, it can be inferred that hormone-regulated molting signals are likely play distinctly different roles in the *S. paramamosain* molting cycle, and these will serve as promising candidates for future analyses.

During early postmolt, *S. paramamosain* still has a soft cuticle and thus may be more vulnerable to bacteria, viruses, or predators^3,40,55^. As with all crustaceans, *S. paramamosain* does not have an adaptive immune system and instead relies upon an innate immune system to avoid exogenous stresses. Antimicrobial peptides (AMPs) are important effectors in innate immunity, and these were up-regulated from PoM to InM in *S. paramamosain*. The AMPs included crustin 1, crustin 4, and ALF 1. Moreover, other transcripts homologous to a number of immune-related genes, including c-type lectin gene families, were identified as differentially expressed during the molting cycle. The functions of these DEGs need to be further confirmed as immune and protective tactics that allow *S. paramamosain* to avoid stress during the molting cycle.

It is well known that crustaceans accumulate nutrients prior to molting to provide enough energy for ecdysis. Various changes in protein, lipid, and carbohydrate content have been detected during the course of the molting cycle in crustaceans^67,68^. In the present study, changes in the lipid composition were found to correspond well with the functions of these lipids during the molting cycle. For example, lipid metabolism-related genes, including lipase 3, alcohol dehydrogenase, and fatty acid-binding protein, were accumulated by the crabs before molting, reached a peak during PrM, and were depleted during PoM. The accumulation of carbohydrates such as glucose and glycogen, which are used primarily as a direct source of metabolic energy, have been observed in several crustacean species prior to the molt^4^. The expression profile of carbohydrate metabolism-related transcripts appears to reflect an increase in the energy requirements of *S. paramamosain* as the molt cycle progresses. The enzymes involved include UDP-glucuronosyltransferase and 6-phosphogluconolactonase. Most of the protein metabolism-related DEGs displayed relatively high expression during InM, followed by a gradual decrease across the rest of the molting cycle. These genes included arginine kinase, glutamine synthetase 2, and glutamate dehydrogenase. In summary, the expression profiles of these transcripts indicate that molting induction creates stress that may impact metabolic function.

In conclusion, we have established a comprehensive transcriptomic repertoire to deduce the molecular events involved in the molting process of *S. paramamosain*. To the best of our knowledge, this is the first systematic transcriptome derived from whole crab bodies, including all the individual organs, during the molting cycle in crustaceans. This transcriptome dataset significantly expands the available genomic information for *S. paramamosain* and provides fundamental support for future research on the molecular mechanisms of the molting cycle in this species. Moreover, the new description of major transcriptional events during different molting stages and the determination of their timing provide temporal markers for future studies of molting progress and regulation in crustaceans.

## Materials and methods

### Experimental design and animal sampling

Healthy juvenile mud crabs from a full-sib family with an average carapace width of 1 cm at growth stage II (Fig. 1) were sampled from this research group’s facility at Ningbo University. These individuals were cultured in tanks with adequate aeration and a temperature of 25 °C, and were supplied with food twice daily. All procedures involving animals throughout the experiments were conducted in strict accordance with the National Institutes of Health guide for the care and use of laboratory animals.

The molting stages of all crabs were determined by examination of pleopod paddles for epidermal retraction, pigmentation, setae development, and the presence of matrix or internal cones in the setal lumen. Molting stages were classified as follows: pre-molt (PrM, Fig. 1A), post-molt (PoM, Fig. 1B), and intermolt (InM-I, InM-II, InM-III, and InM-IV, Fig. 1C–F)^3,4,69^. According to the descriptions and definitions of molting stages in previous studies^70,71^, PoM should correspond to stage B; InM-I, InM-II, InM-III, and InM-IV correspond to stage C, and PrM corresponds to stage D. Three whole crabs were collected from each of these six molting stages and the samples were combined to generate a single sample for sequencing. Three samples were taken as biological replicates for each molting stage. Nine individual crabs were sampled at each molting stage. All samples were immediately frozen and stored in liquid nitrogen until RNA isolation.

### Transcriptome library construction and Illumina sequencing

The total RNA was isolated using the TRIzol method (Invitrogen, USA) according to the manufacturer’s protocol. Total RNA was treated with DNase to remove DNA contamination. The RNA integrity was assessed using the RNA Nano 6000 Assay Kit of the Agilent Bioanalyzer 2100 system (Agilent Technologies, CA, USA). Only high-quality RNA samples (OD260/280 = 1.8–2.2, OD260/230 ≥ 2.0, RIN ≥ 6.5, 28S:18S ≥ 1.0, > 10 µg) were used to construct the sequencing library^72,73^.

A total of 1.5 μg RNA per sample was used as input material for the RNA sample preparation. Sequencing libraries were generated using the NEBNext^®^ Ultra™ RNA Library Prep Kit for Illumina^®^ (NEB, USA) following the manufacturer’s recommendations, and index codes were added to attribute sequences to each sample. First-strand cDNA was synthesized using random hexamer primers, and M-MuLV reverse transcriptase (RNase H-) second-strand cDNA synthesis was subsequently performed using DNA polymerase I and RNase H. After adenylation of the 3′ ends of DNA fragments, NEBNext Adaptor with a hairpin loop structure was ligated to prepare for hybridization. In order to preferentially select cDNA fragments of 250–300 bp in length, the library fragments were purified with an AMPure XP system (Beckman Coulter, Beverly, USA). Then, 3 µl USER Enzyme (NEB, Ipswich, MA, USA) was used with size-selected, adaptor-ligated cDNA at 37 °C for 15 min followed by 5 min at 95 °C before PCR. The PCR was performed with Phusion High-Fidelity DNA polymerase, universal PCR primers, and index (X) primer. Finally, PCR products were purified (AMPure XP system), and library quality was assessed on the Agilent Bioanalyzer 2100 system^72^.

The clustering of the index-coded samples was performed on a cBot Cluster Generation System using a TruSeq PE Cluster Kit v3-cBot-HS (Illumina) according to the manufacturer’s instructions. After cluster generation, the library preparations were sequenced on an Illumina Hiseq platform, and paired-end reads were generated^73^.

### Assembly of sequencing data, gene annotation, and classification

The raw paired-end reads were trimmed and subjected to quality control using SeqPrep (https://github.com/jstjohn/SeqPrep) and Sickle (https://github.com/najoshi/sickle) with default parameters. Then, clean data from the three samples were used to perform RNA de novo assembly using Trinity (http://trinityrnaseq.sourceforge.net/)^74^. All the assembled transcripts were searched against the NCBI (http://www.ncbi.nlm.nih.gov/), NR, Nt, Swissprot (http://www.ebi.ac.uk/uniprot/), KOG (http://www.ncbi.nlm.nih.gov/COG/), KEGG (https://www.kegg.jp/kegg/)^75^ and KO (KEGG Ortholog database, http://www.geneontology) databases. The remaining unmatched unigenes were further analyzed using ESTscan, and the predicted coding sequences were translated into peptide sequences. GATK3 software was used to perform SNP calling. SSRs of the transcriptome were identified using MISA (http://pgrc.ipk-gatersleben.de/misa/misa.html), and the primers for each SSR were designed using Primer3 (http://primer3.sourceforge.net/releases.php).

### Differential gene identification, enrichment, and pathway analysis

The gene expression levels were calculated using FPKM^76^. Differential expression analysis of two samples was performed using the DEGseq (2010) R package. DESeq provides statistical routines for determining differential expression in digital gene expression data. Genes with an adjusted P value < 0.05 found by DESeq were assigned as differentially expressed using a model based on the negative binomial distribution. The P-value was adjusted using the q value^77^. The q value < 0.005 & |log2 (foldchange)|> 1 were set as the threshold criteria for significantly differential expression between adjacent samples (InM-I vs PoM, InM-II vs InM-I, InM-III vs InM-II, InM-IV vs InM-III, PrM vs InM-IV, and PoM vs PrM). Gene Ontology enrichment analysis of the DEGs was implemented by the GO seq R packages-based Wallenius non-central hyper-geometric distribution, which can adjust for gene length bias in DEGs^78^. KOBAS software^79^ was used to test the statistical significance of the enrichment of DEGs in KEGG pathways.

### qRT-PCR analysis

To validate the results from RNA-seq differential gene expression analysis, qRT-PCR was carried out on all six molting stages. Twelve genes from different gene expression clusters were randomly selected for qRT-PCR assays. PCR primers were designed based on the assembled transcriptome sequences (Supplementary Tab. S13). The β-actin gene was amplified in parallel as an internal control.

Total RNA was extracted, and the first-strand cDNA was synthesized by using a PrimeScript RT Reagent Kit with gDNA Eraser (Takara, Kusatsu, Shiga, Japan). All samples were run in triplicate in separate tubes; each cDNA sample was run in duplicate. The qRT-PCR was conducted using SYBR Green Premix Ex Taq (Takara, Kusatsu, Shiga, Japan) in an ABI 7500 Sequence Detection System (Applied Biosystems). A standard curve was initially generated to assess accuracy, and primers with efficiency of amplification between 95 and 105% were chosen for the following qRT-PCR. The comparative Ct method (with the formula 2-ΔΔCt) was used to analyze the expression levels of different genes^80^. Student’s t-test was conducted using SPSS 20.0 (http://www-01.ibm.com/software/analytics/spss/), and P values less than 0.05 were considered statistically significant.

## Supplementary Information

Supplementary Information 1.

Supplementary Information 2.

Supplementary Information 3.

Supplementary Information 4.

Supplementary Information 5.

Supplementary Information 6.

Supplementary Information 7.

Supplementary Information 8.

Supplementary Information 9.

Supplementary Information 10.

Supplementary Information 11.

Supplementary Information 12.

Supplementary Information 13.

Supplementary Information 14.

Supplementary Information 15.

Supplementary Information 16.

Supplementary Information 17.

Supplementary Information 18.

## Data Availability

The datasets generated during the current study were deposited in the National Center for Biotechnology Information (NCBI) Sequence Read Archive (https://www.ncbi.nlm.nih.gov/sra/PRJNA687923) with the accession number PRJNA687923, SRR13347083, SRR13347093, SRR13347084, SRR13347091, SRR13347092, SRR13347088, SRR13347089, SRR13347085, SRR13347082, SRR13347090, SRR13347087, SRR13347080, SRR13347086, SRR13347081, SRR13347077, SRR13347079, SRR13347078 and SRR13347076.
