# Supplementary figures and images for: Whole-body transcriptome analysis provides insights into the cascade of sequential expression events involved in growth, immunity, and metabolism during the molting cycle in Scylla paramamosain
Source: Sci Rep. 2022 Jul 6;12:11395. doi: 10.1038/s41598-022-14783-w (PMC9259733; doi:10.1038/s41598-022-14783-w)

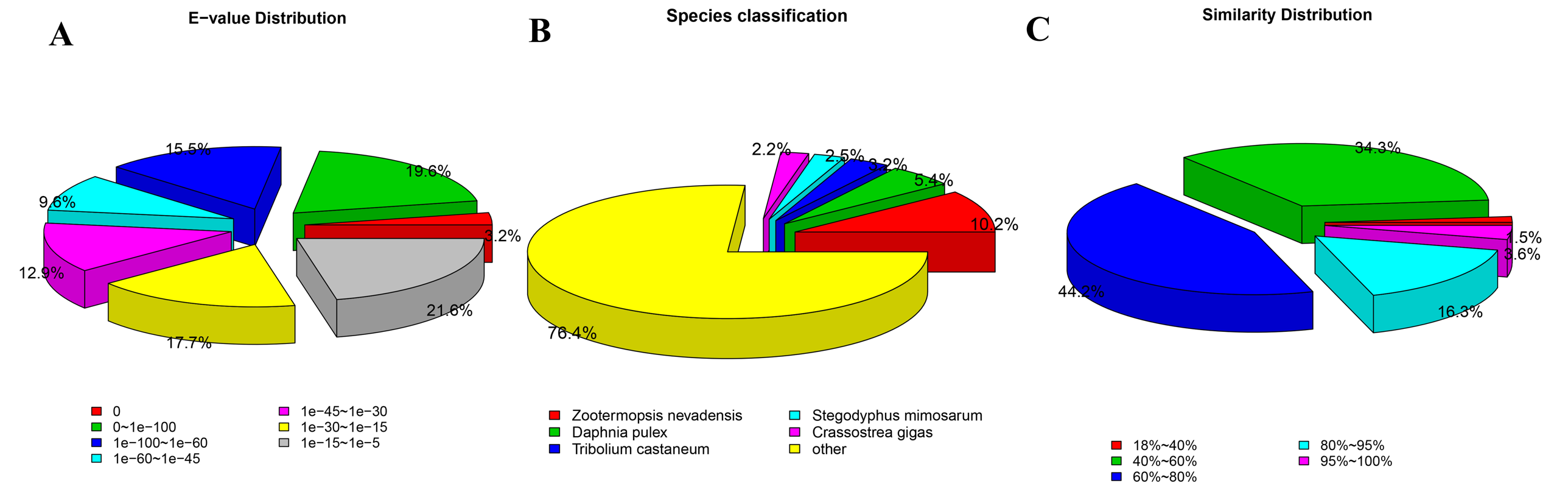

Supplement: Supplementary file 1 — Supplementary Information 1. [file 41598_2022_14783_MOESM1_ESM.jpg]

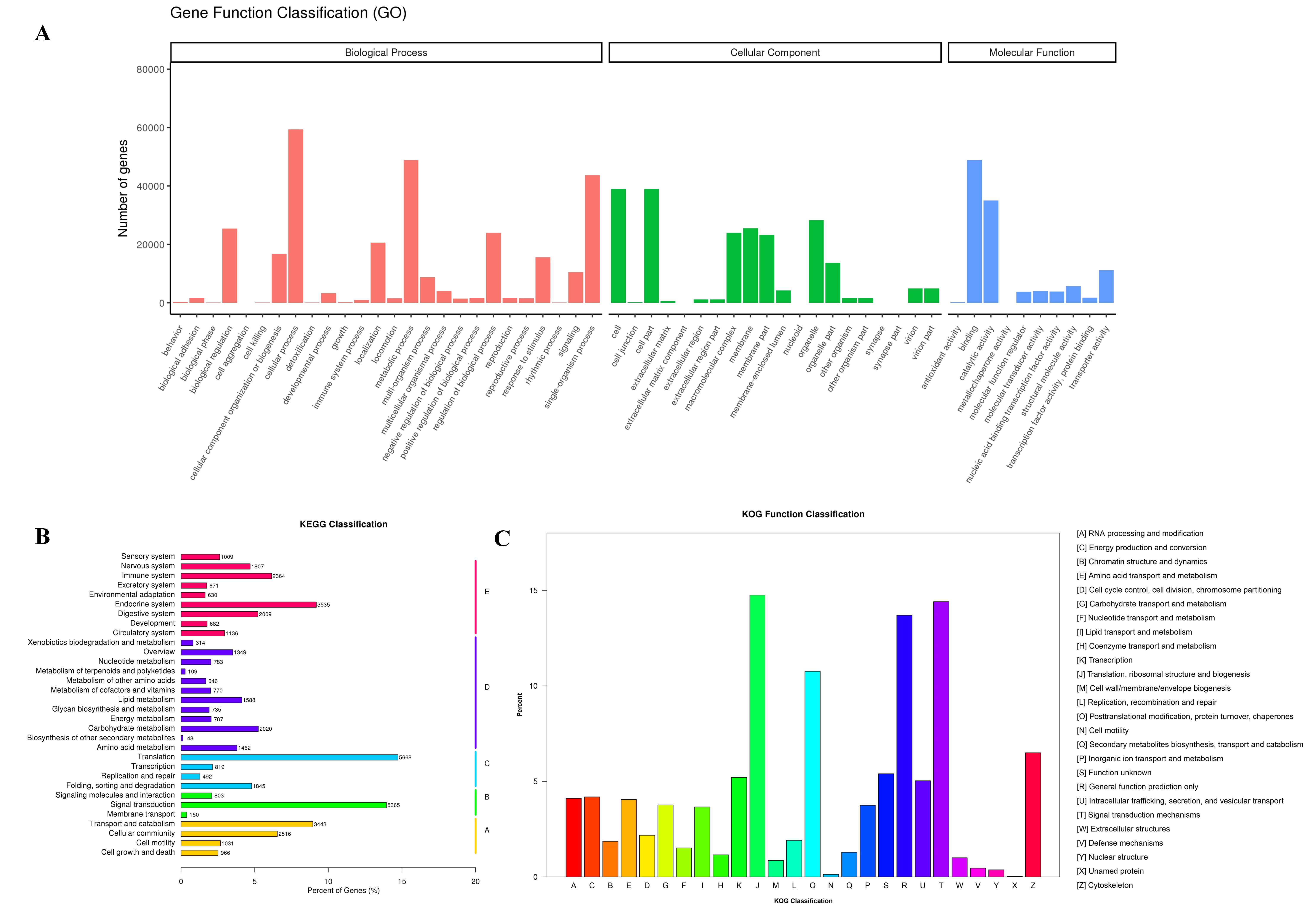

Supplement: Supplementary file 2 — Supplementary Information 2. [file 41598_2022_14783_MOESM2_ESM.jpg]

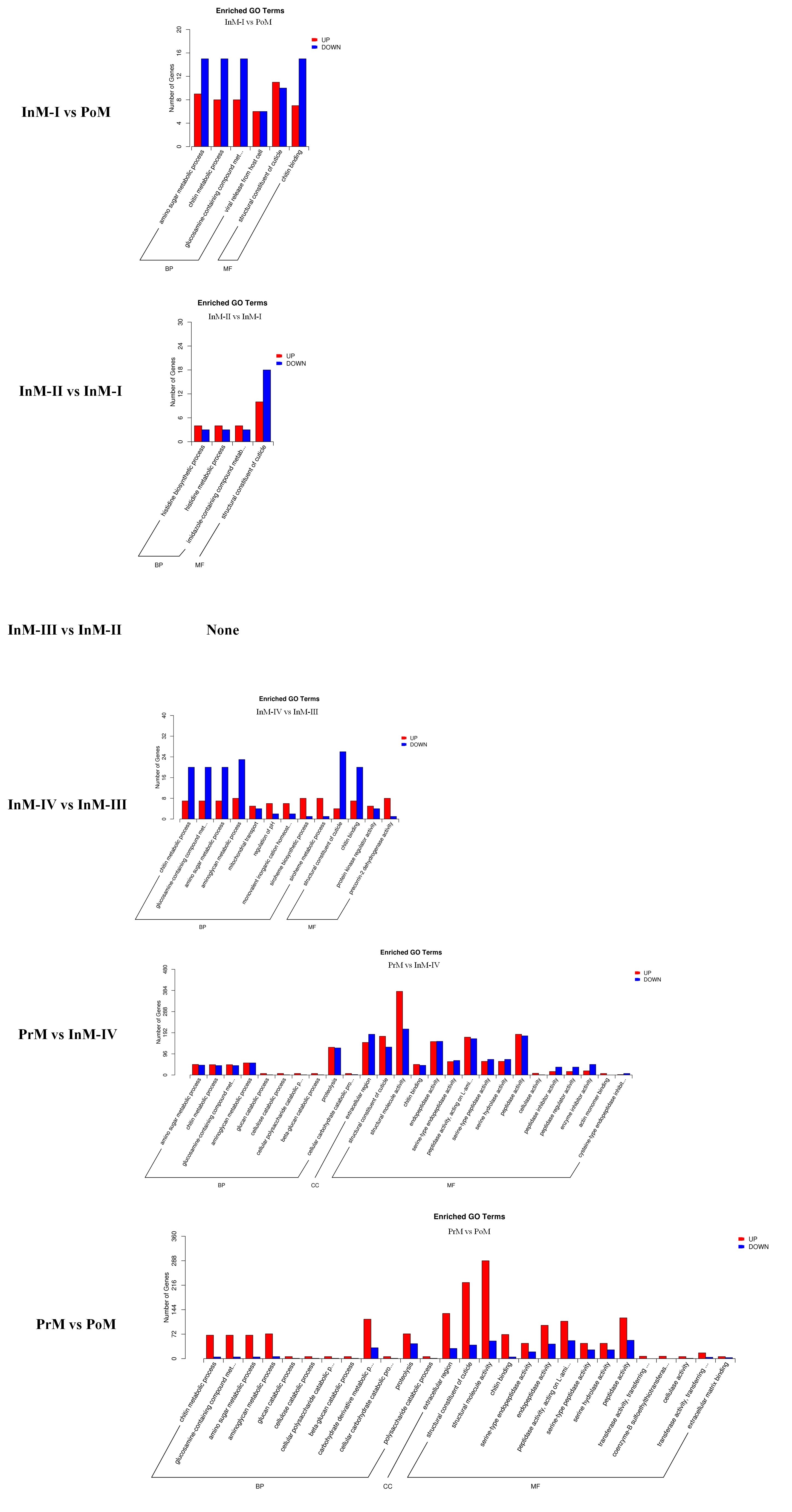

Supplement: Supplementary file 4 — Supplementary Information 4. [file 41598_2022_14783_MOESM4_ESM.jpg]
