# Supplementary material for: Whole-body transcriptome analysis provides insights into the cascade of sequential expression events involved in growth, immunity, and metabolism during the molting cycle in Scylla paramamosain
Source: Sci Rep. 2022 Jul 6;12:11395. doi: 10.1038/s41598-022-14783-w (PMC9259733; doi:10.1038/s41598-022-14783-w)
Supplement: Supplementary file 18 — Supplementary Information 18. [file 41598_2022_14783_MOESM18_ESM.docx]

**Supplementary figure legends**

**Figure S1**. Similarity analysis between *Scylla paramamosain* unigenes and NR database. (A) E-value (<1E-5) distribution of top BLAST hits for each *S. paramamosain* unigene; (B) Top-hit species distribution for BLAST matches for *S. paramamosain* unigenes; (C) Similarity of *S. paramamosain* putative proteins with known proteins in the NR database (>18%).

**Figure S2**. Functional classification and pathway assignment of unigenes of *Scylla paramamosain*. (A) Gene Ontology (GO) classification; (B) Clusters of Orthologous Groups of proteins (KOG) classifications; (C) Kyoto Encyclopedia of Genes and Genomes (KEGG) pathway enrichment.

**Figure S3**. The 22 clusters of 20,436 differentially expressed genes (DEGs) among all the molting stages determined by K-means clustering. The x-axis indicates the molting stage. The y-axis indicates the log2(ratio) of gene expression. Each grey row represents the relative expression of DEGs in a cluster. The blue line represents the average value of all members in one cluster. The red line indicates the reference. Above the red reference line represents up-regulation, and below the line represents down-regulation. The total number within a cluster is shown after each subcluster.

**Figure S4**. Gene ontology enrichment of all differentially expressed genes (DEGs) in six comparisons of adjacent molting stages in *Scylla paramamosain*. The results are summarized in three main GO categories. The x-axis represents the names of these GO subcategories. The y-axis indicates the number of DEGs expressed in a given sub-category.
